# Supplementary material for: Post-marketing quality surveillance of selected antibacterial agents marketed in porous borders; the case of Ethiopia-Sudan-Eritrea border
Source: PLoS One. 2024 Aug 12;19(8):e0308223. doi: 10.1371/journal.pone.0308223 (PMC11318851; doi:10.1371/journal.pone.0308223)
Supplement: S1 Text — (DOCX) [file pone.0308223.s001.docx]

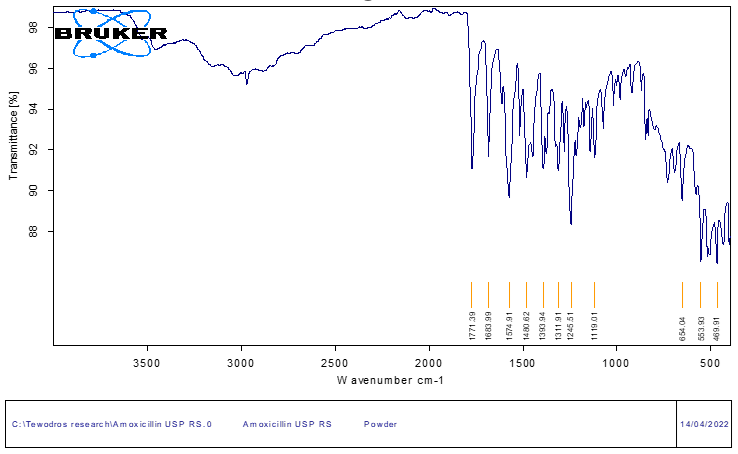

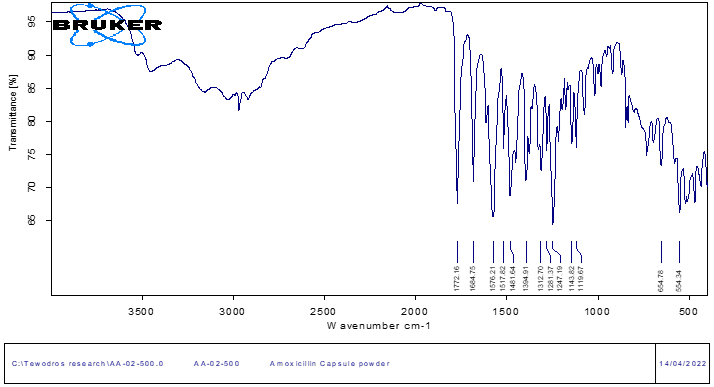


Supplementary Figure 1(A): IR Spectrum of amoxicillin USP RS Figure 1(B): IR Spectrum of amoxicillin sample AA_02_500

Supplementary files: Tables

Supplementary Table 1: Detailed description of samples collected for the study

| **S/_NO_** | **Code** | **Collection site** | **Brand name** | **Bath No** | **Country of origin** | **Manufacturer** |
| --- | --- | --- | --- | --- | --- | --- |
|  | AHG_01_500 | Humera | AMOXID cap 500 | 28228 | Ethiopia | APF PLC |
|  | AK-01_500 | Kokit | Amox – 500 | 706210313 | China | CSPC Shijiazhuang co,.Ltd, |
|  | AAG_01_500 | Abderafi | AMOXID cap 500 | 31423 | Ethiopia | APF PLC |
|  | AM_02_500 | Metema | AMOXID cap 500 | 32490 | Ethiopia | APF PLC |
|  | AMY_01_500 | Mai kadra | AMOXID cap 500 | 32476 | Ethiopia | APF PLC |
|  | AG_01_500 | Gendwuha | Amox-500 | 706201101 | China | CSPC Shijiazhuang Co., Ltd |
|  | AA_02_500 | Abderafi | Amox-500 | 706210316 | China | CSPC Shijiazhuang Co., Ltd |
|  | AMY_02_500 | Mai kadra | Amox-500 | 706210316 | China | CSPC Shijiazhuang Co., Ltd |
|  | AM_01_500 | Metema | Amox-500 | 706210427 | China | CSPC Shijiazhuang co., Ltd |
|  | AK_02_500 | Kokit | Amoxicillin 500mg | 1030391 | Ethiopia | Epharm |
|  | AM_03_500 | Metema | Amoxicillin 500mg | 1030121 | Ethiopia | Epharm |
|  | AH_02_500 | Humera | AMOXID cap 500 | 31074 | Ethiopia | APF PLC |
|  | AG_03_500 | Gendwuha | AMOXID cap 500 | 31286 | Ethiopia | APF PLC |
|  | AHG_02_500 | Humera | Amoxicillin 500mg | 1030431 | Ethiopia | Epharm |
|  | AMY_03_500 | Mai kadra | Amoxicillin 500mg | 1030431 | Ethiopia | Epharm |
|  | AH_01_500 | Humera | Amoxicillin 500mg | 1030431 | Ethiopia | Epharm |
|  | AMYG_01_500 | Mai kadra | Amoxicillin 500mg | 1030391 | Ethiopia | Epharm |
|  | AA_01_500 | Abderafi | AMOXID cap 500 | 26619 | Ethiopia | APF PLC |
|  | AMG_01_500 | Metema | Amoxicillin 500mg | 1030391 | Ethiopia | Epharm |
|  | AG_02_500 | Gendwuha | Amoxicillin 500mg | 1010361 | Ethiopia | Epharm |
|  | AMY_01_250 | Mai kadra | AMYN – 250 | S36420032 | India | KOPRAN LIMITED |
|  | AMYG_01_250 | Mai kadra | AMOXID – 250 | 26506 | Ethiopia | APF PLC |
|  | AG_01_250 | Gendwuha | AMYN – 250 | S36420039 | India | KOPRAN LIMITED |
|  | AAG_01_250 | Abderafi | AMOXID – 250 | 22253 | Ethiopia | APF PLC |
|  | AH_03_250 | Humera | AMOXID – 250 | 29916 | Ethiopia | APF PLC |
|  | AA_01_250 | Abderafi | AMOXID – 250 | 29916 | Ethiopia | APF PLC |
|  | AUMY_01_625 | Mai kadra | INDCLAV 625 | T1150007ET | India | Indchemie Health Specialities Pvt. Ltd. |
|  | AUHG_01_375 | Humera | INDCLAV 375 | T1170001ET | India | Indchemie Health Specialities Pvt. Ltd. |
|  | AUH_02_625 | Humera | INDCLAV 625 | T1150002ET | India | Indchemie Health Specialities Pvt. Ltd. |
|  | AUH_01_625 | Humera | INDCLAV 625 | T1150009ET | India | Indchemie Health Specialities Pvt. Ltd. |
|  | AUAG_01_375 | Abderafi | INDCLAV 375 | T1130019ET | India | Indchemie Health Specialities Pvt. Ltd. |
|  | AUMY_01_375 | Mai kadra | ACINET 375 | 19150567 | India | Alkem labratories Ltd |
|  | AUG_01_625 | Gendwuha | KOACT 625 | EL5019041-C | India | Aurobindo pharma Ltd |
|  | AUAG_01_625 | Abderafi | KOACT 625 | EL5020013-A | India | Aurobindo pharma Ltd |
|  | AUG_02_625 | Gendwuha | CLAVAMYN-625 | K55320019 | India | Kopran limited |
|  | AUHG_01_625 | Humera | KOACT 625 | EL5020013-A | India | Aurobindo Pharma Ltd |
|  | AUA_01_625 | Abderafi | CLAVAMYN-625 | K55320006 | India | KOPRAN LIMITED |
|  | AUMYG_01_625 | Mai Kadra | Clavomid 625 | 92283 | Cyprus | Remedica ltd |
|  | CA_01_500 | Abderafi | AKCIPO 500 | ATB057 | India | Akriti pharmaceuticals pvt., Ltd |
|  | CA_03_500 | Abderafi | Ciproqun 500 | K11420006 | India | KOPRAN LIMITED |
|  | CAG_01_500 | Abderafi | Ciprofloxacin usp 500 | B190807 | China | Zhejiang jingxin pharmaceutical co., ltd |
|  | CG_04_500 | Gendwuha | AKCIPRO 500 | ATB024 | India | Akriti pharmaceuticals pvt., Ltd |
|  | CG_03_500 | Gendwuha | Ciproquin 500 | K11420038 | India | KOPRAN LIMITED |
|  | CG_02_500 | Gendwuha | Ciproquin 500 | K11419108 | India | KOPRAN LIMITED |
|  | CM_01-500 | Metema | CIPRDAC 500 | D21001BY38 | Ethiopia | Cadila Pharmaceuticals Plc |
|  | CM_02_500 | Metema | Ciproquin 500 | K11420008 | India | KOPRAN LIMITED |
|  | CK_01_500 | Kokit | Ciproquin 500 | K11419116 | India | Akriti pharmaceuticals pvt., Ltd |
|  | CK_02_500 | Kokit | AKCIPRO 500 | ATB022 | India | Akriti pharmaceuticals pvt., Ltd, |
|  | CHG_02_500 | Humera | G_CIPROX 500 | HB190201 | Ghana | Guilian Pharmaceutical Co., Ltd |
|  | CH_02_500 | Humera | Ciproflacin tab USP 500 | B190809 | China | Zhejiang Jingxin Pharmaceutical Co., Ltd. |
|  | CH_03_500 | Humera | CIPOFLOX-500 | 203121099 | China | Reyoung Pharmaceuticeutical Co., Ltd |
|  | CM_03_500 | Metema | CIPOFLOX-500 | 193121262 | China | Reyoung pharmaceuticeutical co., Ltd. |
|  | CG_01_500 | Gendwuha | CIPRO-SSP 500 | 00321010010 | Ethiopia | Sansheng Pharmaceutical Plc |
|  | CA_02_500 | Abderafi | CIPRO-SSP 500 | 00321040060 | Ethiopia | Sansheng Pharmaceutical Plc |
|  | CMY_01_500 | Mai kadra | CIPOFLOX-500 | 193121260 | China | Reyoung Pharmaceuticeutical Co., Ltd |
|  | CH_01_500 | Humera | Cipro-SSP 500 | 00320110020 | Ethiopia | Sansheng Pharmaceutical Plc |
|  | CH_04_500 | Humera | Ciproleb-500 | T3579 | India | Leben Laboratories Pvt., Ltd. |
|  | CHG_01_500 | Humera | Cipro-SSP 500 | 00320110070 | Ethiopia | Sansheng Pharmaceutical Plc |
|  | CAG_02_500 | Abderafi | Cipro-SSP 500 | 00320110050 | Ethiopia | Sansheng Pharmaceutical Plc |
|  | CG_01_250 | Gendwuha | Cipro-SSP 250 | 00320120030 | Ethiopia | Sansheng Pharmaceutical Plc |
|  | CAG_01_250 | Abderafi | Cipro-SSP 250 | 00320120020 | Ethiopia | Sansheng Pharmaceutical Plc |
|  | DA_02 | Abderafi | Kodoxy | S55621002 | India | KOPRAN LIMITED |
|  | DA_01 | Abderafi | Doxycap | 26255 | Ethiopia | APF PLC |
|  | DMY_02 | Mai kadra | Doxycap 100 mg | 32221 | Ethiopia | APF PLC |
|  | DH_02 | Humera | Doxyzim 100 | Fbw4a106 | India | Zim Laboratories Limited |
|  | DM_02 | Metema | Doxycap 100 mg | 32219 | Ethiopia | APF PLC |
|  | DMY_03 | Mai kadra | Eapadoxine 100 | Ia206 | Ethiopia | East African Phtical Factory |
|  | DM_01 | Metema | Doxycap 100 mg | 32233 | Ethiopia | APF PLC |
|  | DG_01 | Gendwuha | Doxycap | 26267 | Ethiopia | APF PLC |
|  | DH_01 | Humera | Kodoxy | S55620003 | India | KOPRAN LIMITED |
|  | DMY_01 | Mai kadra | Kodoxy | S55621002 | India | KOPRAN LIMITED |

Supplementary Table 2: Assay value, collection site, brands, and samples of amoxicillin samples

| S/_NO_ | Brand name | Bath No | Manufactured country | Collection site | Code | Assay value (%) - Content Specifications  [92.5%-110.0%] | Conclusion |
| --- | --- | --- | --- | --- | --- | --- | --- |
| 1 | AMOXID cap 500 | 28228 | Ethiopia | Humera | AHG_01_500 | 102.4 | Passed |
| 2 | Amox – 500 | 706210313 | China | Kokit | AK-01_500 | 101.4 | Passed |
| 3 | AMOXID cap 500 | 31423 | Ethiopia | Abderafi | AAG_01_500 | 100.7 | Passed |
| 4 | AMOXID cap 500 | 32490 | Ethiopia | Metema | AM_02_500 | 102.93 | Passed |
| 5 | AMOXID cap 500 | 32476 | Ethiopia | Mai kadra | AMY_01_500 | 103.1 | Passed |
| 6 | Amox-500 | 706201101 | China | Gendwuha | AG_01_500 | 99.85 | Passed |
| 7 | Amox-500 | 706210316 | China | Abderafi | AA_02_500 | 101.55 | Passed |
| 8 | Amox-500 | 706210316 | China | Mai kadra | AMY_02_500 | 101.6 | Passed |
| 9 | Amox-500 | 706210427 | China | Metema | AM_01_500 | 98.34 | Passed |
| 10 | Amoxicillin 500mg | 1030391 | Ethiopia | Kokit | AK_02_500 | 99.7 | Passed |
| 11 | Amoxicillin 500mg | 1030121 | Ethiopia | Metema | AM_03_500 | 97.22 | Passed |
| 12 | AMOXID cap 500 | 31074 | Ethiopia | Humera | AH_02_500 | 101.5 | Passed |
| 13 | AMOXID cap 500 | 31286 | Ethiopia | Gendwuha | AG_03_500 | 102.2 | Passed |
| 14 | Amoxicillin 500mg | 1030431 | Ethiopia | Humera | AHG_02_500 | 103.2 | Passed |
| S/_NO_ | Brand name | Bath No | Manufactured country | Collection site | Code | Assay value (%) - Content Specifications [92.5%-110.0%] | Conclusion |
| 15 | Amoxicillin 500mg | 1030431 | Ethiopia | Mai kadra | AMY_03_500 | 103.8 | Passed |
| 16 | Amoxicillin 500mg | 1030431 | Ethiopia | Humera | AH_01_500 | 102.5 | Passed |
| 17 | Amoxicillin 500mg | 1030391 | Ethiopia | Mai kadra | AMYG_01_500 | 102.1 | Passed |
| 18 | AMOXID cap 500 | 26619 | Ethiopia | Abderafi | AA_01_500 | 102.75 | Passed |
| 19 | Amoxicillin 500mg | 1030391 | Ethiopia | Metema | AMG_01_500 | 99.9 | Passed |
| 20 | Amoxicillin 500mg | 1010361 | Ethiopia | Gendwuha | AG_02_500 | 104.2 | Passed |
| 21 | AMYN – 250 | S36420032 | India | Mai kadra | AMY_01_250 | 102.4 | Passed |
| 22 | AMOXID – 250 | 26506 | Ethiopia | Mai kadra | AMYG_01_250 | 105 | Passed |
| 23 | AMYN – 250 | S36420039 | India | Gendwuha | AG_01_250 | 103.9 | Passed |
| 24 | AMOXID – 250 | 22253 | Ethiopia | Abderafi | AAG_01_250 | 103.8 | Passed |
| 25 | AMOXID – 250 | 29916 | Ethiopia | Humera | AH_03_250 | 100.13 | Passed |
| 26 | AMOXID – 250 | 29916 | Ethiopia | Abderafi | AA_01_250 | 100 | Passed |

Supplementary Table 3: Assay value, collection site, brands, and samples of amoxicillin and clavulanic acid tablets

| S/_NO_ | Brand name | Batch No | Manufactured country | Collection site | Code | Assay value (%) | | Conclusion | |
| --- | --- | --- | --- | --- | --- | --- | --- | --- | --- |
|  |  |  |  |  |  | Amoxicillin | Clavulanate lithium | Amoxicillin | Clavulanate lithium |
| 1 | INDCLAV 625 | T1150007ET | India | Mai kadra | AUMY_01_625 | 94.98 | 98.48 | Passed | Passed |
| 2 | INDCLAV 375 | T1170001ET | India | Humera | AUHG_01_375 | 84.74 | 40.74 | Failed | Failed |
| 3 | INDCLAV 625 | T1150002ET | India | Humera | AUH_02_625 | 94.92 | 89.54 | Passed | Passed |
| 4 | INDCLAV 625 | T1150009ET | India | Humera | AUH_01_625 | 96.46 | 85.82 | Passed | Failed |
| 5 | INDCLAV 375 | T1130019ET | India | Abderafi | AUAG_01_375 | 88.97 | 71.35 | Failed | Failed |
| 6 | ACINET 375 | 19150567 | India | Mai kadra | AUMY_01_375 | 93.82 | 84.50 | Passed | Failed |
| 7 | KOACT 625 | EL5019041-C | India | Gendwuha | AUG_01_625 | 99.83 | 91.28 | Passed | Passed |
| 8 | KOACT 625 | EL5020013-A | India | Abderafi | AUAG_01_625 | 99.81 | 108.78 | Passed | Failed |
| 9 | CLAVAMYN-625 | K55320019 | India | Gendwuha | AUG_02_625 | 97.23 | 105.61 | Passed | Passed |
| 10 | KOACT 625 | EL5020013-A | India | Humera | AUHG_01_625 | 100.30 | 104.36 | Passed | Passed |
| 11 | CLAVAMYN-625 | K55320006 | India | Abderafi | AUA_01_625 | 101.49 | 102.23 | Passed | Passed |
| 12 | Clavomid 625 | 92283 | Cyprus | Mai Kadra | AUMYG_01_625 | 99.33 | 109.38 | Passed | Failed |

Supplementary Table 4: Assay value, collection site, brands, and samples of ciprofloxacin tablet

| S_/NO_ | Brand Name | Batch No | Manufactured country | Collection Site | Code | Assay value (%) | Conclusion |
| --- | --- | --- | --- | --- | --- | --- | --- |
| 1 | AKCIPO 500 | ATB057 | India | Abderafi | CA_01_500 | 87.1 | Failed |
| 2 | Ciproqun 500 | K11420006 | India | Abderafi | CA_03_500 | 88.13 | Failed |
| 3 | Ciprofloxacin usp 500 | B190807 | China | Abderafi | CAG_01_500 | 107.7 | Passed |
| 4 | AKCIPRO 500 | ATB024 | India | Gendwuha | CG_04_500 | 84.8 | Failed |
| 5 | Ciproquin 500 | K11420038 | India | Gendwuha | CG_03_500 | 169.8 | Failed |
| 6 | Ciproquin 500 | K11419108 | India | Gendwuha | CG_02_500 | 92.1 | Failed |
| 7 | CIPRDAC 500 | D21001BY38 | Ethiopia | Metema | CM_01-500 | 90.1 | Failed |
| 8 | Ciproquin 500 | K11420008 | India | Metema | CM_02_500 | 91.3 | Failed |
| 9 | Ciproquin 500 | K11419116 | India | Kokit | CK_01_500 | 91.4 | Failed |
| 10 | AKCIPRO 500 | ATB022 | India | Kokit | CK_02_500 | 88.73 | Failed |
| 11 | G_CIPROX 500 | HB190201 | Ghana | Humera | CHG_02_500 | 92.7 | Passed |
| 12 | Ciproflacin tab USP 500 | B190809 | China | Humera | CH_02_500 | 94.6 | Passed |
| 13 | CIPOFLOX-500 | 203121099 | China | Humera | CH_03_500 | 95.8 | Passed |
| 14 | CIPOFLOX-500 | 193121262 | China | Metema | CM_03_500 | 96.2 | Passed |
| 15 | CIPRO-SSP 500 | 00321010010 | Ethiopia | Gendwuha | CG_01_500 | 95.1 | Passed |
| 16 | CIPRO-SSP 500 | 00321040060 | Ethiopia | Abderafi | CA_02_500 | 95.4 | Passed |
| 17 | CIPOFLOX-500 | 193121260 | China | Mai kadra | CMY_01_500 | 99.2 | Passed |
|  |  |  |  |  |  |  |  |
| S_/NO_ | Brand Name | Batch No | Manufactured country | Collection Site | Code | Assay value (%) | Conclusion |
| 19 | Ciproleb-500 | T3579 | India | Humera | CH_04_500 | 105.54 | Passed |
| 20 | Cipro-SSP 500 | 00320110070 | Ethiopia | Humera | CHG_01_500 | 107.35 | Passed |
| 21 | Cipro-SSP 500 | 00320110050 | Ethiopia | Abderafi | CAG_02_500 | 105.2 | Passed |
| 22 | Cipro-SSP 250 | 00320120030 | Ethiopia | Gendwuha | CG_01_250 | 80.21 | Failed |
| 23 | Cipro-SSP 250 | 00320120020 | Ethiopia | Abderafi | CAG_01_250 | 80.3 | Failed |

Supplementary Table 5: Assay value, collection site, samples of doxycycline

| S.NO | Brand name | Bach no | Manufactured country | Collection site | Code | Assay value (%) | Conclusion content specifications [95.0-105.0%] |
| --- | --- | --- | --- | --- | --- | --- | --- |
| 1 | Kodoxy | S55621002 | India | Abderafi | DA_02 | 91.8 | Failed |
| 2 | Doxycap | 26255 | Ethiopia | Abderafi | DA_01 | 95 | Passed |
| 3 | Doxycap 100 mg | 32221 | Ethiopia | Mai kadra | DMY_02 | 170.1 | Failed |
| 4 | Doxyzim 100 | Fbw4a106 | India | Humera | DH_02 | 99.7 | Passed |
| 5 | Doxycap 100 mg | 32219 | Ethiopia | Metema | DM_02 | 94.3 | Failed |
| 6 | Eapadoxine 100 | Ia206 | Ethiopia | Mai kadra | DMY_03 | 53.6 | Failed |
| 7 | Doxycap 100 mg | 32233 | Ethiopia | Metema | DM_01 | 75.4 | Failed |
| 8 | Doxycap | 26267 | Ethiopia | Gendwuha | DG_01 | 95 | Passed |
| 9 | Kodoxy | S55620003 | India | Humera | DH_01 | 94.6 | Failed |
| 10 | Kodoxy | S55621002 | India | Mai kadra | DMY_01 | 95.2 | Passed |

Supplementary Table 6: Uniformity of weight and disintegration time of amoxicillin samples

| S/_NO_ | Sample code | Mean weight (mg)+/-SD | Acceptance Value (%) | Disintegration time (minutes) ± SD |
| --- | --- | --- | --- | --- |
| 1 | AHG_01_500 | 595.85 ± 0.022 | 6.2 | 5.15 ± 0.21 |
| 2 | AK-01_500 | 577.04 ± 0.0095 | 2.3 | 7.3 ± 0.23 |
| 3 | AAG_01_500 | 595.51 ± 0.0175 | 4.2 | 5.32 ± 0.2 |
| 4 | AM_02_500 | 587.79 ± 0.02 | 6.21 | 6.6 ± 0.31 |
| 5 | AMY_01_500 | 593.84± 0.021 | 6.6 | 7.57 ± 0.33 |
| 6 | AG_01_500 | 589.11± 0.013 | 3.2 | 5.38 ± 0.09 |
| 7 | AA_02_500 | 597.02±0.022 | 5.4 | 6.235 ± 0.36 |
| 8 | AMY_02_500 | 595.33±0.0087 | 2.2 | 5.8 ± 0.408 |
| 9 | AM_01_500 | 599.94±0.0058 | 1.6 | 5.73 ± 0.3 |
| 10 | AK_02_500 | 591.98 ± 0.0097 | 2.340 | 6.2 ± 0.23 |
| 11 | AM_03_500 | 583.80 ±0.0057 | 2.7 | 6.92 ± 0.34 |
| 12 | AH_02_500 | 584.1 ±0.0188 | 4.6 | 6.51 ± 0.5 |
| 13 | AG_03_500 | 588.24±0.014 | 4.1 | 6.4 ± 0.43 |
| 14 | AHG_02_500 | 580.17±0.008 | 3.83 | 5.72 ± 0.43 |
| 15 | AMY_03_500 | 581.65±0.0157 | 6.04 | 6.6 ± 0.3 |
| 16 | AH_01_500 | 580.54 ±0.0102 | 3.5 | 6.31 ± 0.2 |
| 17 | AMYG_01_500 | 582.53±0.0235 | 6.21 | 6.07 ± 0.09 |
| 18 | AA_01_500 | 586.03±0.017 | 5.3 | 6.4 ± 0.43 |
| 19 | AMG_01_500 | 577.09±0.005 | 1.2 | 6.7 ± 0.47 |
| 20 | AG_02_500 | 586.9±0.031 | 10.1 | 7.23 ± 0.205 |
| 21 | AMY_01_250 | 302.71±0.02 | 5.3 | 6.12 ± 0.2 |
| 22 | AMYG_01_250 | 305.43±0.021 | 8.4 | 5.75 ± 0.3 |
| 23 | AG_01_250 | 298.15 ±0.025 | 8.4 | 5.8 ± 0.24 |
| 24 | AAG_01_250 | 294.40±0.04 | 10.99 | 7.2 ± 0.2 |
| 25 | AH_03_250 | 293.88±0.03 | 6.9 | 6.75 ± 0.25 |
| 26 | AA_01_250 | 288.46 ± 0.0242 | 5.805 | 6.3 ± 0.2 |

Supplementary Table 7: Hardness, disintegration, friability, and weight variation of ciprofloxacin, amoxicillin and clavulanate potassium

| S. No. | Sample code | Average hardness (N) (±SD) | Average diameter (N) (±SD) | \| Disintegration time (minutes) ± SD \| \| \| --- \| --- \| \|  \| | % Friability | Mean weight value (mg)+/-SD | Acceptance Value (%) |
| --- | --- | --- | --- | --- | --- | --- | --- | --- | --- | --- |
| 1 | AUMY_01_625 | 204.5 ± 28.4 | 19.9 ± 0.07 | 22.7 ± 0.5 | 0.008 | 1037.41 ± 0.009 | 2.4 |
| 2 | AUHG_01_375 | 227.9±11.8 | 19.03 ±0.02 | 11.7 ± 0.9 | 0.006 | 791.43 ± 0.006 | 58.7 |
| 3 | AUH_02_625 | 244.8 ±15.2 | 19.9 ± 0.05 | 9.5 ± 0.5 | 0.0095 | 1043.21 ± 0.005 | 5.6 |
| 4 | AUH_01_625 | 261.8 ±14.8 | 19.9 ±0.02 | 9.5 ± 0.5 | 0.004 | 1062.68 ± 0.005 | 14.9 |
| 5 | AUAG_01_375 | 200.7 ± 12.6 | 19 ± 0.04 | 24.3 ± 0.5 | 0.0063 | 784.78 ± 0.007 | 28.8 |
| 6 | AUMY_01_375 | 244.2 ±24.4 | 20.2 ±0.05 | 25.7 ± 2.5 | 0.005 | 776.58 ± 0.009 | 17.0 |
| 7 | AUG_01_625 | 233.9 ± 8.2 | 20.2 ±0.05 | 21.5 ± 0.9 | 0.002 | 1018.24 ± 0.006 | 1.5 |
| 8 | AUAG_01_625 | 258.8 ± 10.6 | 20.2 ±0.02 | 22.5 ± 0.5 | 0.008 | 1017.43 ± 0.008 | 1.9 |
| 9 | AUG_02_625 | 339.7 ± 21.6 | 19.7 ± 0.03 | 48.3 ± 0.5 | 0.009 | 1036.42 ± 0.017 | 5.1 |
| 10 | AUHG_01_625 | 206.4 ± 13.2 | 20.02 ± 0.06 | 6.3 ± 0.5 | 0.003 | 1016.92 ± 0.011 | 3.5 |
| 11 | AUA_01_625 | 253.9 ± 28.7 | 19.8 ±0.04 | 28.5 ± 1.1 | 0.005 | 1021.76 ± 0.009 | 2.2 |
| 12 | AUMYG_01_625 | 356.6 ±18.8 | 21.7 ±0.03 | 41.3 ± 2.5 | 0.003 | 1133.37 ± 0.011 | 2.7 |

Supplementary Table 8: Hardness, disintegration, friability, and weight variation of ciprofloxacin samples

| S. No. | Sample code | Average hardness (N) (±SD) | Average diameter (N) (±SD) | \| Disintegration time (minutes) ± SD \| \| --- \| \|  \| | % Friability | Mean weight value (mg)+/-SD | Acceptance value (%) |
| --- | --- | --- | --- | --- | --- | --- | --- | --- | --- |
| 1 | CA_01_500 | 272.2 ± 20.9 | 16.954 ± 1.2 | 3.5 ± 0.5 | 0.3 | 703.08 ± 0.009 | 13.7 |
| 2 | CA_03_500 | 225 ± 12.5 | 16.548 ± 0.06 | 5.3 ± 0.5 | 0.08 | 645.7 ± 0.007 | 12 |
| 3 | CAG_01_500 | 167 ± 11.9 | 18.102 ±0.025 | 2.3 ± 0.5 | 0.03 | 784.4050 ± 0.021 | 11.3 |
| 4 | CG_04_500 | 288.4 ± 12.4 | 17.404 ± 0.03 | 3.3 ± 0.5 | 0.05 | 705.3630 ± 0.011 | 16.2 |
| 5 | CG_03_500 | 189.1 ± 11.2 | 16.447 ± 0.03 | 7.66 ± 0.5 | 0.08 | 633.6910 ± 0.009 | 15.9 |
| 6 | CG_02_500 | 196.4 ± 3.7 | 16.519 ± 0.02 | 3.33 ± 0.5 | 0.05 | 640.1860 ± 0.008 | 8.3 |
| 7 | CM_01-500 | 216.8 ± 21.8 | 16.446 ± 0.03 | 3.7 ± 0.75 | 0.008 | 664.9430 ± 0.018 | 12.7 |
| 8 | CM_02_500 | 207.9 ± 7.8 | 16.529 ± 0.03 | 5.8 ± 0.4 | 0.05 | 638.7760 ± 0.006 | 9.9 |
| 9 | CK_01_500 | 203.7 ± 8.4 | 16.526 ± 0.03 | 4 ± 0.82 | 0.02 | 644.8990 ± 0.007 | 8.7 |
| 10 | CK_02_500 | 290.3 ± 6.9 | 17.344 ± 0.03 | 3.83 ± 0.85 | 0.02 | 705.2390 ± 0.034 | 17.9 |
| 11 | CHG_02_500 | 194.2 ± 7.4 | 17.819 ± 0.04 | 9.33 ± 0.74 | Broken | 618.5200 ± 0.006 | 7.2 |
| 12 | CH_02_500 | 152.5 ± 22.6 | 17.816 ± 0.94 | 1.7 ± 0.24 | 0.05 | 796.4470 ± 0.013 | 7.1 |
| 13 | CH_03_500 | 108.8 ± 15.9 | 18.13 ± 0.02 | 13.6 ± 0.54 | 0.02 | 780.7860 ± 0.015 | 5.8 |
| 14 | CM_03_500 | 93.4 ± 9.02 | 18.106 ± 0.03 | 3.2 ± 0.24 | Broken | 795.5490 ± 0.009 | 4.6 |
| 15 | CG_01_500 | 116.7 ±14.0 | 18.122 ± 0.01 | 0.72 ± 0.3 | 0.015 | 806.9210 ± 0.016 | 7.4 |
| 16 | CA_02_500 | 132.8 ± 7.2 | 18.074 ± 0.02 | 1.4 ± 0.08 | 0.015 | 807.1380 ± 0.047 | 14.5 |
| 17 | CMY_01_500 | 86 ± 9.6 | 18.117 ± 0.02 | 2.5 ± 0.0 | Broken | 791.5970 ± 0.013 | 3.1 |
| 18 | CH_01_500 | 120.4 ± 3.9 | 18.059 ± 0.02 | 0.625 ± 0.3 | 0.24 | 808.7850 ± 0.024 | 12.3 |
| 19 | CH_04_500 | 196.7 ± 12.53 | 16.9 ± 0.01 | 1.325 ± 0.23 | 0.05 | 696.0050 ± 0.025 | 10.1 |
| 20 | CHG_01_500 | 196.7 ±12.5 | 16.924 ± 0.01 | 1.33 ± 0.01 | 0.05 | 817.3100 ± 0.011 | 8.5 |
| 21 | CAG_02_500 | 154.3 ± 11.3 | 18.146 ± 0.02 | 0.465 ± 0.015 | 0.04 | 818.1850 ± 0.025 | 10.3 |
| 22 | CG_01_250 | 145.7 ± 16.9 | 18.067 ± 0.03 | 0.43 ± 0.03 | 0.08 | 400.1 ± 0.004 | 19.4 |
| 23 | CAG_01_250 | 98.2 ± 5.8 | 11.012 ± 0.01 | 0.5 ± 0.03 | 0.06 | 401.1 ± 0.005 | 19.3 |

Supplementary Table 9: Uniformity of weight and disintegration time of different doxycycline samples

| S/_NO_ | Sample code | Mean weight (mg)+/-SD | acceptance Value (%) | Disintegration time (minutes) ± SD |
| --- | --- | --- | --- | --- |
| 1 | DA_02 | 242.04 ± 0.02 | 8.01 | 8.3 ± 0.24 |
| 2 | DA_01 | 223.00± 0.014 | 10.11 | 6.75 ± 0.25 |
| 3 | DMY_02 | 227.44 ± 0.04 | 78.04 | 8.13 ±1.44 |
| 4 | DH_02 | 249.47 ± 0.031 | 7.4 | 5.65 ± 0.4 |
| 5 | DM_02 | 224.34 ± 0.02 | 8.21 | 5.9 ± 0.3 |
| 6 | DMY_03 | 129.64± 0.012 | 47.9 | 11.42 ± 0.34 |
| 7 | DM_01 | 229.34 ± 0.0214 | 28.3 | 6.5 ± 0.41 |
| 8 | DG_01 | 226.83 ± 0.025 | 9.6 | 6.3 ± 0.4 |
| 9 | DH_01 | 243.01 ± 0.0335 | 11.9 | 7.3 ± 0.3 |
| 10 | DMY_01 | 240.12 ± 0.02 | 7.8 | 7.2 ± 0.2 |
