# Supplementary material for: Post-marketing quality surveillance of selected antibacterial agents marketed in porous borders; the case of Ethiopia-Sudan-Eritrea border
Source: PLoS One. 2024 Aug 12;19(8):e0308223. doi: 10.1371/journal.pone.0308223 (PMC11318851; doi:10.1371/journal.pone.0308223)
Supplement: Attachment — Submitted filename: November 29okpoint by point response.docx [file pone.0308223.s002.docx]

November 16,2023

Response to the decision Letter:

1-Please provide rationale for selecting the sample sites. How and why you select the sites? Detail methodology is mandatory.

**Ans: The sites were selected as potential areas of cross -border trade that includes medicines that enter without proper regulatory (National medicines Authority) oversight. This statement hd been included in the revision ( Please look to line 115-117)**

2-Inside experimental, Please write two headings 1) Instrumentation 2) Chemicals. Provide the source of all items and % purity of chemical used. Please provide details on the validation of instrument. Yes or No. If Yes then who validated the instruments? line 129-144 has been written poorly and several source missing. Kindly read quality manuscript on how to write the details. The source details are mandatory. The country name is not enough to justify.

**Ans: Instrumentation, chemicals with two heading and the necessary details had been included easily in the revision. (Look at lines 125-140)**

**We had used the national medicines authority laboratory an ISO 17025 accredited ones and these are routine procedures they abide for compliance and the details of who and when can be easily provided in the manuscript if needed. The sentences mentioning this fact had been included in the revision (line126-128)**

3- The study design is missing such as sampling methodology, sample size calculation. The current methodology need to improve before we procceed. Include the heading of study design and explain each items.

**Ans: The study design followed WHO recommendations for post marketing quality surveillance protocol and this had been enumerated in the revision easily**. (line 126-128)

4- The experimental details with reference were missing such as Assay methods.

 Ans: I**t is either USP/BP based on the specific medicine label claim and this can be easily incorporated in the revisions.**

5- There is no rational on the selection of the United States Pharmacopoeia 2020? Why? It is necessary to provide the details. There is no sampling methodology, we do not know the product follows which standard (BP/USP). We cannot generalized the results on one standard.

**Ans: Standards were selected based on the Medicines label claim of compliance and all were not analyzed in one standard.  We had used BP/USP standards accordingly. we can easily clarify it on the revisions.**

**Thank you for the important critical inputs and scientific justifications**

sincerely,

Ayenew Ashenef ( on behalf of the authors)
