# Supplementary material for: Post-marketing quality surveillance of selected antibacterial agents marketed in porous borders; the case of Ethiopia-Sudan-Eritrea border
Source: PLoS One. 2024 Aug 12;19(8):e0308223. doi: 10.1371/journal.pone.0308223 (PMC11318851; doi:10.1371/journal.pone.0308223)
Supplement: Attachment — Submitted filename: Rebuttal letter.docx [file pone.0308223.s004.docx]

April 29, 2024

Dear Editor

PLOS one:

Subject: Point by Point response to comments by the editor and reviewer’s (PONE-D-23-33682R1) “Post-marketing Quality Surveillance of Selected Antibacterial Agents Marketed in Porous Borders; the case of Ethiopia-Sudan-Eritrea border”.

First We want to acknowledge the editor and the anonymous reviewers for kind consideration of our manuscript and the important in puts forwarded to us in-order to improve our manuscript.

Here follows point by point response to the comments:

1. Editor’s comments:
a. Journal’s style: The revised manuscript is based on the journals requirement style.
b. Data availability: The data supporting the manuscript’s writing content and conclusion are included in the manuscript and additional data is availed as supplementary information.
c. Acknowledgement and Funding: The manuscript is revised on such comments. Funding and acknowledgements are now separate.
d. Images are now prepared as jpeg file and uploaded
e. Figure 1 map is constructed using qgis 3 software which is in the public domain. httpS://www.qgis.org/en/site

Reply to reviewer 1:

1. General comments;

General comments
• The Authors are appreciated for using an ISO 17025 qualified laboratory for conducting the quality test.

Answer: Thank you for the appreciation!!!
• There are grammatical errors and punctuation marks that need to be addressed throughout the manuscript and I recommend proof-reading of the manuscript again.

Answer; Attempts were cautiously performed to correct the grammatical and punctuation errors in the revisions.

• Use terms consistently throughout the manuscript. For example the words medicine Vs drug, falsified Vs counterfeit. Better to remove the word counterfeit from the article.

Answer: The word medicine and Substandard and falsified medicines had been used throughout the text in the revised manuscript.

• Contesting/disputing issues such as locations, administrative demarcation on the study area, or use of unofficial demarcation of territories in scientific studies is not encouraged. Better to be silent or indicate in neutral way.

Answer: Such issues were addressed in the revisions in a neutral way.

• The National Regulatory Authority in Ethiopia has a guideline for PMS including data collection; did you use that or any other model for your study? Describe this.

Answer; Yes, it had been used.
Specific comments
1. Title of the study (line 1-2)
• Delete the phrase ‘’Ethiopia side of’’.
• Replace ; by :
• Rephrased title: Post-marketing Quality Surveillance of Selected Antibacterial Marketed in Porous Borders: the Case of Ethiopia-Sudan-Eritrea border.

Answer: title was modified per the suggestion
Abstract (lines 25-51)
• Though the abstract contains all the contents, it is good to structure it into background, objectives, Methods, Results and Conclusions.

Answer; Abstract is modified per the suggestions and presented in structured way. ( Lines …..)
• Better to replace the phrase ‘low-quality medicines’ to poor-quality medicines’

Answer : it is replaced in such manner ( line…)
• Line 31, delete corridor

Answer; corridor is deleted (line…)
• Lines 35-36, use the correct name (i.e. replace electronic registration Information system(ERIS) database by Electronic Regulatory Information System (eRIS).

Answer: It is addressed per the suggestion
• Line 38 reads as 15.49% (11/71) were registered but the license period had expired. Have you checked the current application status in the eRIS? This comment also applies to the result section.

Answer: All experiments were done in the time period described in the method. Currently it is not updated considering the time of publication of the manuscript. But the general regulatory procedure is registered products will be availed in the eRIS system before marketing which is applicable to new products but for medicinal products that need license re-newal, it may apply in partial terms.
• Line 47, do you think the conclusion is appropriate. During the study period, there was war/conflict in the area which might be open for illegal distribution of medicines. It is wise to adjust the conclusion considering the situation.

Answer: yes, conflict aggravates the distribution of Substandard and falsified(SF) medicines. The Conclusion is modified as “The study had indicated that poor-quality antibacterial medicines are circulating in the study sites. Therefore, even if the area were affected by conflict at the time of the study, the regulatory bodies should focus on enforcing the necessary measures by collaborating with the regional and national regulatory medicine agencies to ensure that antibacterial medicines availed meet the required mandatory minimum standards.

.”
 
From the 114 health institutions available, how many of them did you include in the data collection process? Please, make it clear.
• What is your sample size and how do you calculate it? The authors should clarify and describe it.
• Sampling procedure is not clearly outlined. How do you select your sampling sites from the existing sites? In addition, describe your inclusion and exclusion criteria for both the medicines to be collected and the sampling sites?
• The authors should describe the sampling technique.

Answer: The sampling procedure is convenient method. Generally, a formula to calculate sample size in this type of studies i.e. medicine quality surveys had not been set up that is agreed among the experts in the field although in some studies sample size calculations employed for hypothesis testings are also deployed and reported in few medicine quality studies publications.

The sampling strategy and procedure had been clearly demonstrated in the revision in the form of diagrammes that is included in the revision (please look at lines 133-140)

The inclusion and exclusion criteria is

Inclusion: Different brands and batches at the site of collection of the selected medicines in the survey. Availability of the sampled medicines in the survey

Exclusion : when the sample amounts are not sufficient for the study parameters . Non availability
• Line 160, correct it as commented earlier.
• Line 230: describe why the authors use excel sheet for the data management and analysis?
Results and discussion
• Line 257: do you think, the reason is weak regulation during release at the port? The illegal medicines might enter to the country through other channels which were unknown to the regulatory authority. Instead of using the word release, it is better to use enter or crossing the border.

Answer: Both lines 160, 230 and 257 had been addressed. It is corrected per the suggestion
• Line 259: It is a concerning issue for the Ethiopian Medicine Regulatory Agency (MRA). Does this statement value to your results and discussion? Better to remove it.

Answer: it is removed per the suggestion.
• In your results and discussion, it is wise to remove the use of falsified medicines. The authors did not encounter falsified medicines. Example line 263.

Answer; It is removed.
• Lines 261-263, better remove it. It is known and practiced that if this illegal practice is known or encountered by the authority, strict actions has been taken.

Answer: It is removed per the suggestion
• Line 268: only 7.89% (3/38) of the imported samples were not registered. What if these three products are authorized by the authority though they are not register? In Ethiopian law, there are circumstances that unregistered but authorized medicine may enter to the market of Ethiopia. Example donation. The Authors should consider the circumstances that are allowed by the Ethiopian law or you need to review the laws and reviser your results about the issue of registration of medicines. This also include for the locally produced medicines.

Answer: a statement about donation had been included in the revision.
• For the samples failed to meet the standards in the identification, assay, disillusion, disintegration, etc., tests; what is the reason behind for the failure? It is good to indicate the possible reasons (i.e. failure of manufacturing, storage, transportation, or other reasons).

Answers: Reasons behind failures were included in the revision at different appropriate places in each results and discussion section.
Conclusions
• Line 388: make it conclusions
• Lines 389-392: already stated in the result section. Adjust the conclusions accordingly.
• Line 393: replace the regulatory authority (EFDA) by the Ethiopian Food and Drug Authority and the region regulatory bodies

Answer; The new conclusion had taken such comments in to consideration.

Response to reviewer 2:

Comments by reviewer 2 are positive feed backs for most of the questions on the check list and for few it is N/A thus it is not needed to add any point by point responses to the comments. We want to say thank you for the positive feed backs.

Reviewer #2: The introduction is well written and states clearly the matter at hand.

Method

The materials and methods used have been well elaborated

Answer: Thanks for the comments

Results and Discussion

Visual inspection
"Hence, it had to strengthen the regulation system, especially in the prone areas where the study sites are located"
Consider changing had to has

Answer: It is addressed per the suggestion.

For most of the subsections under results and discussion they appear to be only results. Please consider discussing the subheadings as well.

Answer: Discussion points with the results had been incorporated in the revision. Thank you for the comments.

**Sincerely,**

**Ayenew Ashenef**

**On behalf of the authors.**
