# Supplementary material for: Post-marketing quality surveillance of selected antibacterial agents marketed in porous borders; the case of Ethiopia-Sudan-Eritrea border
Source: PLoS One. 2024 Aug 12;19(8):e0308223. doi: 10.1371/journal.pone.0308223 (PMC11318851; doi:10.1371/journal.pone.0308223)
Supplement: Attachment — Submitted filename: July 12 response to reviewers second version.docx [file pone.0308223.s005.docx]

**July 12, 2024**

**Rebuttal letter**

**Dear**

**Obed Kwabena Offe Amponsah, PharmD, Ph.D.
Academic Editor
PLOS ONE**

First and foremost, thank you for due consideration of our manuscript PONE-D-23-33682R2

Post-marketing Quality Surveillance of Selected Antibacterial Agents Marketed in Porous Borders; the case of Ethiopia-Sudan-Eritrea border. We want to acknowledge the scientific in puts kindly offered by the anonymous reviewer’s that helped us to improve our manuscript to best scientific standards.

Below, please look at the point by point replies to the reviewer’s comments:

**Comments to the Author**

1. If the authors have adequately addressed your comments raised in a previous round of review and you feel that this manuscript is now acceptable for publication, you may indicate that here to bypass the “Comments to the Author” section, enter your conflict of interest statement in the “Confidential to Editor” section, and submit your "Accept" recommendation.

Reviewer #1: All comments have been addressed

Reviewer #2: All comments have been addressed

**Answer: Thank you for confirming our best attempt to address the previous comments**

2. Is the manuscript technically sound, and do the data support the conclusions?

The manuscript must describe a technically sound piece of scientific research with data that supports the conclusions. Experiments must have been conducted rigorously, with appropriate controls, replication, and sample sizes. The conclusions must be drawn appropriately based on the data presented.

Reviewer #1: Yes

Reviewer #2: Yes

Answer: Thanks

3. Has the statistical analysis been performed appropriately and rigorously?

Reviewer #1: Yes

Reviewer #2: N/A

Answer: Thanks

4. Have the authors made all data underlying the findings in their manuscript fully available?

The [PLOS Data policy](http://www.plosone.org/static/policies.action#sharing) requires authors to make all data underlying the findings described in their manuscript fully available without restriction, with rare exception (please refer to the Data Availability Statement in the manuscript PDF file). The data should be provided as part of the manuscript or its supporting information, or deposited to a public repository. For example, in addition to summary statistics, the data points behind means, medians and variance measures should be available. If there are restrictions on publicly sharing data—e.g. participant privacy or use of data from a third party—those must be specified.

Reviewer #1: Yes

Reviewer #2: Yes

Answer: All data used to wrote the manuscript is included in the body of the paper as well as as supplementary files.

5. Is the manuscript presented in an intelligible fashion and written in standard English?

PLOS ONE does not copyedit accepted manuscripts, so the language in submitted articles must be clear, correct, and unambiguous. Any typographical or grammatical errors should be corrected at revision, so please note any specific errors here.

Reviewer #1: Yes

Reviewer #2: Yes

Answer: The manuscript had been revised to address typographical and grammatical errors thus it fulfills the standard scientific English writing.

6. Review Comments to the Author

Please use the space provided to explain your answers to the questions above. You may also include additional comments for the author, including concerns about dual publication, research ethics, or publication ethics. (Please upload your review as an attachment if it exceeds 20,000 characters)

Reviewer #1: All comments are addressed as per the recommendation. The authors should still conduct topographic and grammatical checks. It is worth to mention one important issues to be addressed. No falsified medicine was encountered during the assessment and it is good to indicate this in the result and conclusion part of the manuscript.

Answer; Grammatical and typographical issues had been addressed extensively. Per the recommendation, the conclusion had included the above statement (Line …)

Reviewer #2: The manuscript looks better now.
However, some of your references are rather too old.

Reference 1 although very old appears to support a historic fact and this is allowed.
References 8, 9 and 11 however are over 10 years old and do not accurately reflect currents status of the situations as cited in the manuscript. In most of these cases recent literature are available, consider replacing or citing alongside more recent literature.

Answer; Per the recommendations the above references more than 10 years old had been replaced by recent references containing similar facts as the outdated references. Please look at lines 452ff,457ff, 467ff …………………………………

7. PLOS authors have the option to publish the peer review history of their article ([what does this mean?](https://journals.plos.org/plosone/s/editorial-and-peer-review-process#loc-peer-review-history)). If published, this will include your full peer review and any attached files.


If you choose “no”, your identity will remain anonymous but your review may still be made public.


**Do you want your identity to be public for this peer review?** For information about this choice, including consent withdrawal, please see our [Privacy Policy](https://www.plos.org/privacy-policy).

Reviewer #1: No

Reviewer #2: No

Answer: From the authors perspective, we declared that the reviewer’s ideas with our responses to the scientific comments in anonymized manner can be published among the different revision versions.

With best regards,

Ayenew Ashenef,

On Behalf of the Authors
