# Supplementary material for: Post-marketing quality surveillance of selected antibacterial agents marketed in porous borders; the case of Ethiopia-Sudan-Eritrea border
Source: PLoS One. 2024 Aug 12;19(8):e0308223. doi: 10.1371/journal.pone.0308223 (PMC11318851; doi:10.1371/journal.pone.0308223)
Supplement: Attachment — Submitted filename: Reviewer comments (PONE-D-23-33682_R1).docx [file pone.0308223.s003.docx]

**Reviewer comments (PONE-D-23-33682R1)**

**General comments**

- The Authors are appreciated for using an ISO 17025 qualified laboratory for conducting the quality test.
- There are grammatical errors and punctuation marks that need to be addressed throughout the manuscript and I recommend proof-reading of the manuscript again.
- Use terms consistently throughout the manuscript. For example the words medicine Vs drug, falsified Vs counterfeit. Better to remove the word counterfeit from the article.
- Contesting/disputing issues such as locations, administrative demarcation on the study area, or use of unofficial demarcation of territories in scientific studies is not encouraged. Better to be silent or indicate in neutral way.
- The National Regulatory Authority in Ethiopia has a guideline for PMS including data collection; did you use that or any other model for your study? Describe this.

**Specific comments**

1. **Title of the study (line 1-2)**

- Delete the phrase ‘’Ethiopia side of’’.
- Replace ; by :
- Rephrased title: Post-marketing Quality Surveillance of Selected Antibacterials Marketed in Porous Borders: the Case of Ethiopia-Sudan-Eritrea border.

**Abstract (lines 25-51)**

- Though the abstract contains all the contents, it is good to structure it into background, objectives, Methods, Results and Conclusions.
- Better to replace the phrase ‘low-quality medicines’ to poor-quality medicines’
- Line 31, delete corridor
- Lines 35-36, use the correct name (i.e. replace electronic registration Information system(ERIS) database by Electronic Regulatory Information System (eRIS).
- Line 38 reads as 15.49% (11/71) were registered but the license period had expired. Have you checked the current application status in the eRIS? This comment also applies to the result section.
- Line 47, do you think the conclusion is appropriate. During the study period, there was war/conflict in the area which might be open for illegal distribution of medicines. It is wise to adjust the conclusion considering the situation.

**Introduction**

- The introduction has coherence problem. Some concepts that are not aligned with this study (example disease burden) should be removed. The justification should also be stated in one paragraph. It should not be stated here and there (lines 84-85, line 97…, line 99 ,,,, etc.
- Lines 72-73, proper regulation includes both continuous post-marketing surveillance, and fast regulatory actions. Either delete both continuous post-marketing surveillance and fast regulatory actions or focus on ‘post-marketing surveillance’.
- Lines 75-77 states about the burden of infectious disease. This is another concept which does not align with the research objective. You can mention it as a problem that can be exacerbated by substandard and falsified products.
- Lines 81-85; care should be taken here. 1). Are you sure about the absence of comprehensive evaluation of basic regulatory functions, 2). I do not think, it is worthy to mention about maturity level in this study. Better to remove lines 81-83. Line 84 and 85 are enough to justify for your study.
- Line 87, replace with national drug policy or use the correct name.
- Align lines 81-82, and the last paragraph of the introduction section.
- Lines 90-94, better to indicate in the method section
- Seriously review lines 98-104 - repetition of terms and concepts.
- Line 107 is repetition. It is stated earlier in the introduction section

**Methods and Materials**

- Properly describe the method section
- The sample size is very small
- Line 117: Both zones are located in the Amhara regional state of Ethiopia. This is a contesting issue. Better to to remove.
- From the 114 health institutions available, how many of them did you include in the data collection process? Please, make it clear.
- What is your sample size and how do you calculate it? The authors should clarify and describe it.
- Sampling procedure is not clearly outlined. How do you select your sampling sites from the existing sites? In addition, describe your inclusion and exclusion criteria for both the medicines to be collected and the sampling sites?
- The authors should describe the sampling technique.
- Line 160, correct it as commented earlier.
- Line 230: describe why the authors use excel sheet for the data management and analysis?

**Results and discussion**

- Line 257: do you think, the reason is weak regulation during release at the port? The illegal medicines might enter to the country through other channels which were unknown to the regulatory authority. Instead of using the word release, it is better to use enter or crossing the border.
- Line 259: It is a concerning issue for the Ethiopian Medicine Regulatory Agency (MRA). Does this statement value to your results and discussion? Better to remove it.
- In your results and discussion, it is wise to remove the use of falsified medicines. The authors did not encounter falsified medicines. Example line 263.
- Lines 261-263, better remove it. It is known and practiced that if this illegal practice is known or encountered by the authority, strict actions has been taken.
- Line 268: only 7.89% (3/38) of the imported samples were not registered. What if these three products are authorized by the authority though they are not register? In Ethiopian law, there are circumstances that unregistered but authorized medicine may enter to the market of Ethiopia. Example donation. The Authors should consider the circumstances that are allowed by the Ethiopian law or you need to review the laws and reviser your results about the issue of registration of medicines. This also include for the locally produced medicines.
- For the samples failed to meet the standards in the identification, assay, disillusion, disintegration, etc., tests; what is the reason behind for the failure? It is good to indicate the possible reasons (i.e. failure of manufacturing, storage, transportation, or other reasons).
- Please review the terms such as registration, re-registration, authorization from the legal context of Ethiopia

**Conclusions**

- Lines 389-392: already stated in the result section. Adjust the conclusions accordingly.
- Line 393: replace the regulatory authority (EFDA) by the Ethiopian Food and Drug Authority and the region regulatory bodies
